# Molecular Dynamics Simulations of PtTi High-Temperature Shape Memory Alloys Based on a Modified Embedded-Atom Method Interatomic Potential

**DOI:** 10.3390/ma15155104

**Published:** 2022-07-22

**Authors:** Jung Soo Lee, Young-Bum Chun, Won-Seok Ko

**Affiliations:** 1 Industrial Science and Technology Research Institute, Inha University, Incheon 22212, Korea; jslee1318@inha.ac.kr; 2 Advanced Material Development Division, Korea Atomic Energy Research Institute, Daejeon 34057, Korea; 3 Department of Materials Science and Engineering, Inha University, Incheon 22212, Korea

**Keywords:** molecular dynamics, shape memory alloy, Platinum–Titanium, martensitic phase transformation, modified embedded-atom method

## Abstract

A new second nearest-neighbor modified embedded-atom model-based PtTi binary interatomic potential was developed by improving the pure Pt unary descriptions of the pre-existing interatomic potential. Specifically, the interatomic potential was developed focusing on the shape memory-associated phenomena and the properties of equiatomic PtTi, which has potential applications as a high-temperature shape memory alloy. The simulations using the developed interatomic potential reproduced the physical properties of the equiatomic PtTi and various intermetallic compound/alloy compositions and structures. Large-scale molecular dynamic simulations of single crystalline and nanocrystalline configurations were performed to examine the temperature- and stress-induced martensitic transformations. The results show good consistency with the experiments and demonstrate the reversible phase transformation of PtTi SMA between the cubic B2 austenite and the orthorhombic B19 martensite phases. In addition, the importance of anisotropy, constraint and the orientation of grains on the transformation temperature, mechanical response, and microstructure of SMA are presented.

## 1. Introduction

The significance and requirement of more efficient and better performing machines or technologies capable of producing higher energy output increased in the ever-growing energy demand and consumption era. Constant research and development of materials capable of performing at high temperatures and in extreme environments are needed to keep up with this progress. Currently, there is a plethora of high-temperature structural alloys commercially available, such as the Ni-based superalloys [1,2,3,4,5]. Nevertheless, new systems of high-temperature alloys that exhibit superior properties to those of the commercial superalloy counterparts are also being developed, e.g., the γ-γ’ Co-based superalloys [6,7], and refractory high-entropy alloys [8,9,10,11,12]. On the other hand, these alloys are limited to structural applications, i.e., their alloy designs do not account for functional properties, such as the shape memory effect, thermoelectric effect, superconductivity, and magnetocaloric effect. Hence, the need for functional materials, such as shape memory alloys (SMAs), capable of working at high operating temperatures increased [13,14,15].

SMAs are alloys that exhibit a shape memory effect (ability to restore their original shape upon heating) and superelasticity (ability to withstand large strains elastically) [16,17]. SMAs are used for various applications, such as dental devices, bone plates, glass frames, actuators, and stents [13,17,18,19,20,21,22]. On the other hand, the most widely used Ni–Ti-based SMAs have a temperature limit of about 373 K, owing to their low phase transformation temperature range [23]. Accordingly, high-temperature shape memory alloys (HTSMAs) that can recover their shapes at higher temperatures were developed by alloying the Ni–Ti system with Hf, Pd, Pt, Au, and Zr [13,14,15,24,25,26,27,28,29,30,31,32,33], or through the exploration of new alloy systems (e.g., Pd–Ti and Pt–Ti) [34,35,36,37,38].

The HTSMAs are anticipated to have good applicability as components, but the most promising and prospective high-temperature applicability are the solid-state actuators because they can respond to the temperature changes and generate mechanical responses [23]. Using SMA solid-state actuators, more compact, lightweight, and higher energy density actuator systems can be built and installed than pneumatic, hydraulic, and motor-driven ones [23]. Several examples show the actual application and potential use of high-temperature shape memory alloys, primarily, but not limited to, aerospace sectors, such as the active clearance control actuation of a turbofan engine [39], helical actuators for surge control in helicopter engine compressors [40], variable geometry chevrons, springs, and wires for jet engines or lunar surface applications [41], micro-flow effectors, and the nosecone tilting actuators of missiles [42,43].

A rigorous understanding of the alloys and their related response and phenomena are essential before the HTSMAs of various alloy systems can be fully exploited and utilized. On the other hand, the experiments often become complicated because the phenomena associated with phase transformation and twinning are atomic-scale processes. Furthermore, extra complexity is added to the experiments because the investigation and analysis of the HTSMAs must be performed at elevated temperatures. In such scenarios, atomistic simulation techniques, such as density functional theory (DFT) calculations and molecular dynamics (MD), could well support the experimental observations and even discover new attributes and their origins. For instance, numerous investigations employing large-scale MD simulations have effectively revealed origins to several phenomena [44,45,46,47,48,49,50,51,52,53,54,55,56,57,58,59,60,61,62,63,64,65,66,67]. For an MD simulation, an interatomic potential for the system of interest is essential, but the performance of the potential is another crucial aspect that is dependent on the model it is based on and the parameter fitting. Hitherto, there is a lack of interatomic potentials that can reliably simulate phase transformations in SMAs, and the number of available interatomic potentials further dwindles in the case of HTSMAs.

Therefore, this study developed a new Pt–Ti binary interatomic potential based on the second nearest-neighbor modified embedded-atom method (2NN MEAM) model [68,69,70] over an existing interatomic potential in order to establish one that is suitable for simulating a shape memory alloy because the Pt–Ti system exhibits one of the highest phase transformation temperatures (1273–1363 K) [71] between austenite and martensite. The equiatomic PtTi SMA transforms from cubic B2 austenite to orthorhombic B19 martensite and vice versa. The reason for the development of the interatomic potential based on the 2NN MEAM model is because the most critical aspect of an SMA, i.e., the martensitic transformation between B2 austenite and B19 martensite, can be well captured and replicated using the implicitly provided angle-dependent potential parameters that reflect the directionality of the atomic bonds [68,69,70]. This model successfully established interatomic potentials for other SMA systems (NiTi [72] and PdTi [73]), and its extensions to ternary interatomic potentials were used to analyze SMA composites [49,67].

The reference database for fitting and optimizing the interatomic potential parameters were generated via DFT calculations. A thorough evaluation of the developed interatomic potential was conducted by reproducing the properties of several alloy compositions and structures. The martensitic transformations of the single and nanocrystalline structures of equiatomic PtTi SMAs were demonstrated via thermal and mechanical loading cycles. These simulations show that the developed Pt–Ti binary interatomic potential can be used to simulate and analyze the SMA aspect of the equiatomic PtTi and investigate the properties of other compositions and configurations.

## 2. Development of Interatomic Potential

### 2.1. DFT Database Construction

The required energy and force database for the optimization of potential parameters were established by performing ab initio DFT calculations. All calculations were performed using the VASP (Vienna ab initio simulation package) code [74,75,76]. The projector-augmented wave (PAW) method [77] was employed in the DFT framework within the local density approximation (LDA) and generalized gradient approximation (GGA) of the Perdew–Burke–Ernzerhof (PBE) form [78]. The relevant PAW potential for the pure Ti was chosen to treat 3*p* electrons as a part of the valence. The Methfessel–Paxton smearing method with 0.1 eV width was used for all calculations. For the plane wave basis set, a cutoff energy value of 400 eV was used, and a *k*-point mesh of 21 × 21 × 21 was employed for the body-centered cubic (bcc) and face-centered cubic (fcc) and primitive unit cells. The similar *k*-point density was applied to simulation cells with the hexagonal close-packed (hcp) structure, intermetallic compound structures, and defect structures.

The defect properties were calculated by configuring supercells with 108 and 96 atoms (in pristine states) for the fcc and hcp structures, respectively, and relaxing the positions of all the atoms in a supercell at constant cell shape and volume. The convergence criteria for the total energy of a cell and force on atoms were set to 10^−6^ eV and 10^−2^ eV/Å, respectively.

The solute migration energy was determined using the nudged elastic band (NEB) method [79,80]. The surface-related properties were obtained by configuring 11.2–13.8 Å thick rectangular slabs with a vacuum region of approximately 10 Å. The calculations of phonon-related properties were conducted using the Phonopy code [81,82], with convergence criteria for energy and force of 10^−8^ eV and 10^−4^ eV/Å, respectively.

The energies and forces related to structures at finite temperatures were determined via a two-step DFT calculation. Initially, the configurations at finite temperatures were derived by performing ab initio MD simulations using rough convergence criteria (single *k*-point and default cutoff energy). The calculations with higher accuracy were performed using an increased *k*-point mesh and cutoff energy (400 eV) for the final energies and forces.

### 2.2. 2NN MEAM Interatomic Potential Parameter Optimization

Database construction and parameter fitting for the pure unary system was carried out first to develop Pt–Ti binary interatomic potential. In this work, only the pure Pt unary interatomic potential parameters were optimized. The pure Ti unary interatomic potential parameters were adopted from a previous study [72] based on optimization using the force matching process. A previous work used a 2NN MEAM pure Pt interatomic potential [69], but the present study improvised the fitting quality by incorporating a DFT calculation with a force matching process. A 2NN MEAM potential requires fourteen independent parameters to be established. Of these fourteen, four parameters were involved in the universal equation of state: cohesive energy ($E_{c}$), equilibrium nearest-neighbor distance ($r_{e}$), reference structure bulk modulus (B), and adjustable parameter (d). Seven parameters are essential for the electron density: four decay lengths ($\beta^{\left(0\right)}$*,* $\beta^{\left(1\right)}$*,* $\beta^{\left(2\right)}$*,* and $\beta^{\left(3\right)}$) and three weighting factors ($t^{\left(1\right)}$*,* $t^{\left(2\right)}$*,* and $t^{\left(3\right)}$). Two $C_{min}$ and $C_{max}$ parameters are essential for many-body screening. Finally, parameter *A* is necessary for the embedding function. A more detailed description of the 2NN MEAM pure element potential parameters can be found elsewhere [68,69,70].

A feedback loop process was performed to optimize the Pt unary potential parameters, i.e., the energies and forces predicted by the temporary potential parameters were compared with those of the DFT calculations, and a genetic algorithm was used to deduce optimal parameters in order to minimize the errors for the energy and force. The fcc structure was chosen as a reference structure because fcc is the most stable structure of Pt under ambient conditions. Of the three cohesive energies of fcc Pt, 5.77, 7.044, and 5.526 eV/atom, which were obtained experimentally [83] and calculated by DFT (present work via local density approximation (LDA) and GGA), the experimental value was used for parameter fitting. Table 1 lists the final optimized Pt unary interatomic potential parameters.

The Pt–Ti binary system was optimized based on these unary interatomic potential parameters. For this, 13 additional parameters, necessary to describe a binary system, were established (for detailed information on the parameters, readers are referred to the reference [70,84]). In the case of the binary system potential parameters, only the energy-related atomic configurations and the physical properties of intermetallic compounds were considered without the force-related information for optimization as fitting targets. This was done because the required flexibility for the force matching process was not provided by the 2NN MEAM model for the binary system potential parameters [72].

The binary Pt–Ti system consists of several intermetallic compounds: Pt_8_Ti, Pt_3_Ti, Pt_5_Ti_3_, PtTi, and PtTi_3_ [85]. Of these intermetallic compounds, the equiatomic PtTi and its off-stoichiometric compositions exhibit a martensitic phase transformation between a high-temperature stable cubic B2 austenite and low-temperature stable orthorhombic B19 martensite. As the present Pt–Ti interatomic potential was aimed for the application and analysis of Pt–Ti binary SMAs that involve a martensitic transformation, a higher priority was set toward reproducing the physical properties related to the equiatomic PtTi. In addition, the configurations of other intermetallic compounds (Pt_8_Ti, Pt_3_Ti, Pt_5_Ti_3_, and PtTi_3_) were also included to enable the interatomic potential to simulate more diversified structures, compositions, and physical conditions. The binary solid solution phases, i.e., Pt-rich fcc and Ti-rich hcp structures, were only considered for the validation and transferability evaluation and not for parameter fitting.

Table 2 lists the finalized interatomic potential parameters for the Pt–Ti binary system. For this potential, a radial cutoff distance of 4.5 Å was confirmed to be adequate for simulating the phase transformation phenomena of the targeted alloy compositions and structures with acceptable computing efficiency. Therefore, all the molecular static and dynamic simulations in this work were carried out using that cutoff distance.

## 3. Performance of the Developed 2NN MEAM Potential

The thoroughly examined performance of the developed interatomic potential (unary and binary) is presented in this section. Various physical properties were obtained using the developed interatomic potential and those from DFT calculations, and experiments were carried out. LAMMPS code [86] was employed to calculate the properties using the developed interatomic potential. Unless specified otherwise, molecular static simulations were performed with a minimum of 4000 atoms per cell, and full relaxation of the cell dimensions and atomic positions were allowed. For MD simulations, a timestep of 2 fs was used.

### 3.1. Performance of the Pure Pt Unary Interatomic Potential

**Figure 1:**
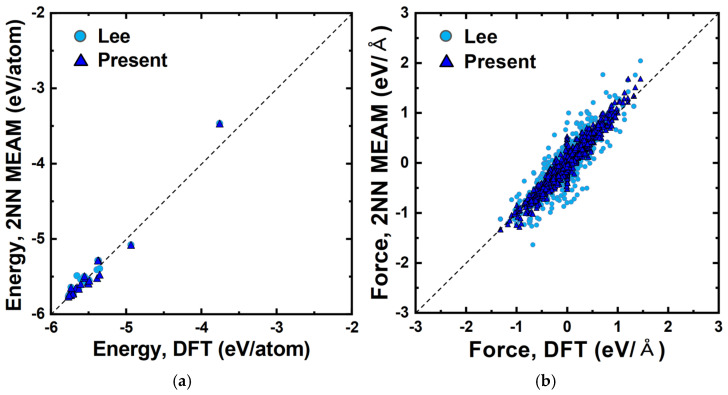
Scatter plots of (**a**) energies and (**b**) forces, demonstrating the fitting accuracies with respect to the present DFT calculation. The values obtained using the present and previous (Ref. [69]) 2NN MEAM interatomic potentials for pure Pt are compared. The dashed lines represent ideal correlations between the 2NN MEAM potentials and the DFT calculation.

This section reports the accuracy and transferability of the developed pure Pt interatomic potential. First, the static energy and force statistical correlation analysis of atomic configurations used for parameter fitting was conducted between the results obtained using the developed potential and those predicted by the present DFT calculation to demonstrate the accuracy of force matching. Figure 1a,b shows the graphical representation of the analysis of energy and force, respectively. The accuracy of the predicted energies and forces of atomic configurations predicted by the present 2NN MEAM potential appears to be improved somewhat compared to the previous 2NN MEAM potential [69], but it is more important to determine if the potential can reproduce the specific physical properties. Listed in Table 3 are the structural, elastic, and defect properties at 0 K, predicted using the developed potential and those from other various sources and methods (DFT, experiment [69,83], and previous interatomic potential [69]). Although not remarkably accurate, both previous [69] and the present interatomic potentials reproduce those DFT-calculated and experimental properties satisfactorily.

**Figure 2:**
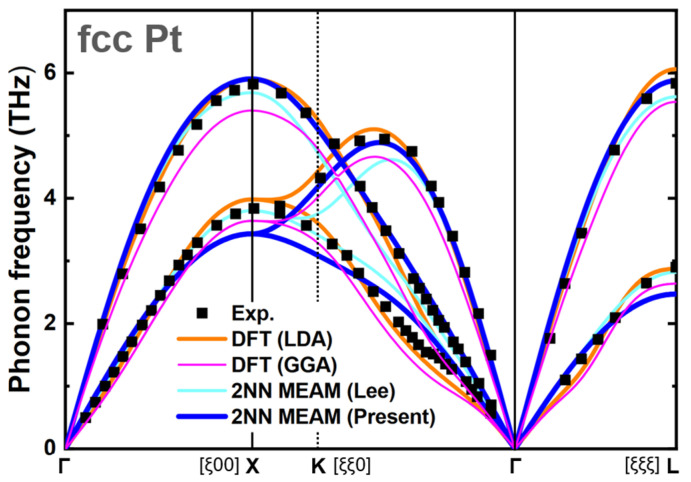
Phonon spectra of fcc Pt predicted based on the harmonic approximation. The spectra obtained from the experiment [88], the present DFT calculation, and the present and previous [69] 2NN MEAM potentials are compared.

**Figure 3:**
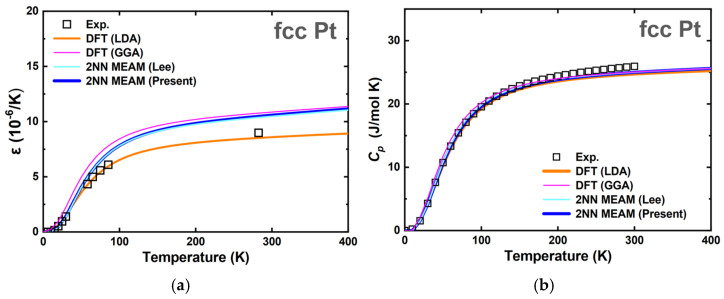
Thermal properties (**a**) thermal expansion coefficient (ε) and (**b**) heat capacity ($C_{P}$) of fcc Pt predicted based on the quasiharmonic (QH) approximation. The curves obtained from the experiment [89,90], the present DFT calculation (LDA and GGA), and the present and previous [69] 2NN MEAM potentials are compared.

For an interatomic potential to be used under a wide range of conditions, it is crucial to reproduce the properties at 0 K and simulate those at finite temperatures. The good reproducibility and accordance were also extended to the physical properties at finite temperatures. Table 4, Figure 2 and Figure 3 present a range of finite temperature properties, such as the melting temperature ($T_{m}$), enthalpy of melting ($\Delta H_{m}$), thermal expansion (ε), and heat capacity ($C_{P}$). Melting-related simulations were performed using an isobaric-isothermal (*NPT*) ensemble. The interface method, which simulates with the simultaneous presence of solid and liquid phases, was adopted to determine the melting temperature. At the melting temperature and zero pressure, independent simulations of liquid and solid phases were carried out to obtain $\Delta H_{m}$ and the volume change upon melting. The melting properties were verified only with the experimental values because of the difficulty in calculating the melting-related properties via the DFT method. The comparison showed that the $T_{m}$ and $\Delta H_{m}$ values obtained using the present potential were closer to those of the experiment. Quasi-harmonic approximation was used for ε and $C_{P}$. The results shown in Figure 2 and Figure 3 show good agreement with the DFT and experimental values. The adequacy of the developed pure Pt interatomic potential in reproducing the various properties can be confirmed by these comparisons and verifications. Hence, this Pt unary potential, in conjunction with the previously developed Ti unary potential [72], can be extended to construct the Pt–Ti binary interatomic potential.

### 3.2. Performance of the Pt–Ti Binary Interatomic Potential

The physical properties of intermetallic binary compounds were calculated to examine the reproducing performance of the Pt–Ti binary interatomic potential. Table 5 lists the structural parameters (lattice parameters and atomic volume), bulk modulus, and enthalpy of formation of intermetallic compounds. The comparison of several properties for various intermetallic compounds obtained via different methods (experiment [85,91], DFT (LDA and GGA)), and using the present interatomic potential showed good accuracy in fitting and prediction. A clear difference in the enthalpy of formation between the cubic B2 (stable at higher temperatures) and orthorhombic B19 (stable at lower temperatures) structures of PtTi can be observed, which may be related to the martensitic transformation of the phases. As the structures of the phases differ during the martensitic transformation, a change in atomic volume tends to occur naturally. This difference in atomic volume with high accuracy, with respect to the DFT calculation, could also be seen from the calculated results. In this case, shrinkage occurs in the forward transformation from austenite (B2) to martensite (B19).

**Figure 4:**
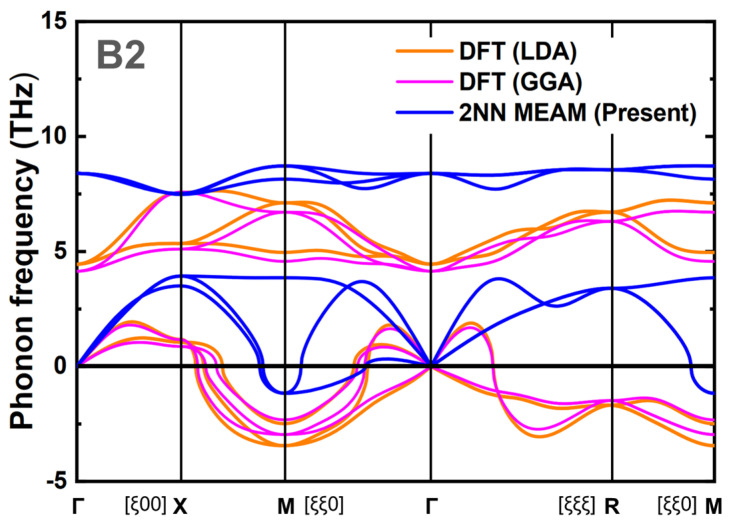
Phonon spectra of B2 structure PtTi predicted based on the harmonic approximation. The spectra obtained from the present DFT calculation (LDA and GGA), and the present 2NN MEAM potential are compared.

The performance of the present Pt–Ti binary interatomic potential, in terms of its ability to reproduce the martensitic phase transformation, was evaluated further by examining the phonon properties. The phonon instability of the B2 structure is particularly important for the occurrence of a phase transformation, as reported in previous DFT calculations [72,92]. These studies reported that the martensitic phase transformation from the austenite to martensite structure is correlated with the occurrence of the imaginary phonon branches in the phonon spectrum of the B2 cubic structure. For example, the present DFT calculations predict the occurrence of instability along the directions near the [*ξξ*0] and [*ξξξ*] points, as shown in Figure 4. The prediction by the present binary potential showed a similar tendency, while the overall frequencies were higher than those predicted by the DFT calculations. In particular, the developed binary potential reproduced the imaginary phonon branch along the direction near the [*ξξ*0] point, which is closely related to the transformation into the B19 martensite structure [72,92]. Therefore, the developed binary potential should reproduce the occurrence of the martensitic transformation. The demonstration of the phase transformation behavior using the developed interatomic potential by performing additional MD simulations will be presented in the next section (Section 4).

**Figure 5:**
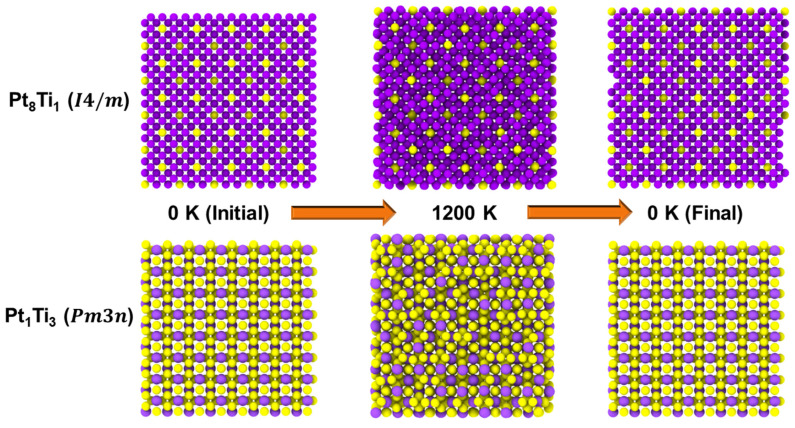
Atomic configurations of $D1_{a}$-Pt_8_Ti and A15-PtTi_3_ intermetallic compounds at 0 K (before heating), 1200 K (after heating), and 0 K (after cooling). The purple and yellow atoms represent Pt and Ti, respectively.

Although the primary focus was on the martensitic transformation and its features of equiatomic PtTi, other intermetallic compound (Pt_8_Ti, $L1_{2}$-Pt_3_Ti, $D0_{24}$-Pt_3_Ti, Pt_5_Ti_3_, and PtTi_3_) properties were also in good agreement. In addition to the stable intermetallic compounds that exist in the phase diagram [85], the hypothetical $P\bar{3}m1$-Pt_3_Ti structure was also included when calculating the properties. Even for these structures, the physical properties could be well reproduced using the present interatomic potential. Nevertheless, in most cases, the predicted enthalpy of formation of intermetallic compounds showed a positive deviation with respect to the DFT results. This suggests that during the simulation of intermetallic compounds, their stability could pose a problem, or undesirable/unexpected phase transformations could occur. Therefore, thermal cycle MD simulations from 0 K to 1200 K and back to 0 K were carried out on the selected intermetallic compounds. Detailed thermal cycle simulation results and analysis involving equiatomic PtTi B2 and B19 structures will be discussed later in the next section (Section 4.1). Figure 5 shows the atomic configurations of *D*1*_a_*-Pt_8_Ti and *A*15-PtTi_3_ at 0 K and 1200 K during thermal cycling. Pt_8_Ti maintains its structure for the entire simulated temperature range, unlike PtTi_3_, which loses its crystallinity at higher temperatures. Thus, the present potential is unsuitable for simulating the structures related to PtTi_3_, and care should be taken when simulating other intermetallic structures to avoid possible instability issues.

Finally, the transferability of the present potential was scrutinized by inspecting the properties of solid solutions. In addition to the dilute heat of the solution, the defect-related energies, such as the vacancy–solute binding energy, solute–solute binding energy, and solute migration energy, were also considered. The calculated properties of both Pt-rich fcc and Ti-rich hcp structures are shown in Table 6. The dilute heat of solution is defined as the change in enthalpy associated with the solute atom in a solid solution structure without solute–solute interaction. For example, the dilute heat of the Pt-rich fcc solid solution ($E_{d}^{Ti}$) was calculated using following equation:

(1) $$E_{d}^{Ti}=E_{N}^{PtTi}-E_{N-1}^{Pt}-E_{1}^{Ti}$$

Here, $E_{N}^{PtTi}$ is the total energy of a supercell calculated when (*N* − 1) Pt atoms and one Ti atom exist in a fcc solid solution, $E_{N-1}^{Pt}$ is the total energy of a fcc supercell with (*N* − 1) Pt atoms, and $E_{1}^{Ti}$ is the energy of one Ti atom in its reference state (hcp). Regarding the dilute heat of solutions, the present potential showed reasonable performance. The binding energy results, which are defined as the energy difference between two defects or solutes when they are near and far from each other, appear to show varying degrees of accuracy. An attractive interaction is represented by a positive binding energy and vice versa. The binding energies in the case of Pt-rich fcc were well predicted in general, except for the second nearest-neighbor Ti–Ti interaction. By contrast, the Ti-rich hcp binding energies showed relatively higher deviations with respect to the DFT results. On the other hand, the DFT results (between LDA and GGA methods) showed considerable differences. The solute energy, which is the barrier associated with the solute atom migration to an adjacent vacancy position, was overestimated for both solid solutions. Nevertheless, care must be taken while performing simulations for investigations that are related to these properties with deviating predictions.

## 4. Applications of the Developed Interatomic Potential

As in the previous section, the developed potential was shown to reproduce the physical properties. The current section reports a few possible applications using the developed potential via MD simulations. The developed interatomic potential was validated to appropriately simulate the reversible phase transformation between the austenite (B2) and the martensite (B19), which is one of the key phenomena for the working mechanism of the targeted alloy system. Hence, further investigations with varying conditions were carried out.

### 4.1. Temperature-Induced Phase Transformation Simulation of Single Crystal Equiatomic PtTi SMAs

**Figure 6:**
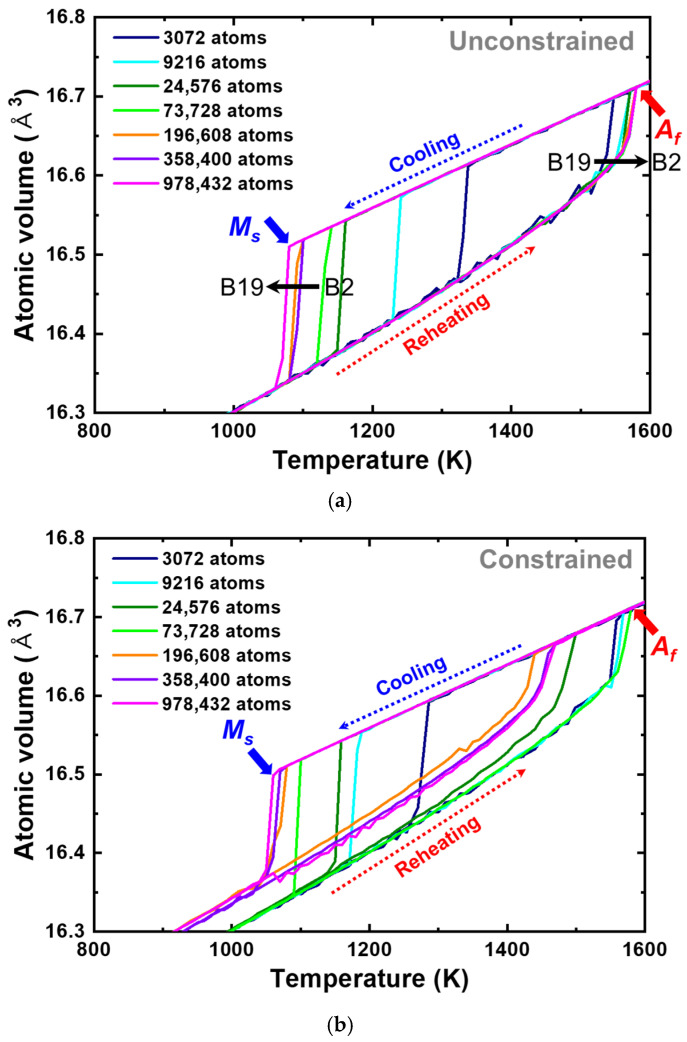
Atomic volume vs. temperature plots of single crystalline equiatomic PtTi with (**a**) unconstrained and (**b**) constrained conditions predicted using the present interatomic potential. The number of atoms of individual cell configurations is provided in legends. The cooling and reheating curves are indicated using dotted lines. The onset of the abrupt change in the atomic volume during cooling (occurring due to an austenite to martensite phase transformation) is marked by the martensite start temperature ($M_{s}$), and the offset of the abrupt change in atomic volume during reheating (occurring due to martensite to austenite phase transformation) is marked by the austenite finish temperature ($A_{f}$).

The first application of the present interatomic potential was implemented on the equiatomic PtTi SMAs by varying the temperature and studying the shape memory effect. Different sizes of single crystal cells, from 3072 to 978,432 atoms with austenite (B2) structures, were configured to analyze the size effect. The temperature and pressure were controlled using the Nosé–Hoover thermostat and barostat [93,94] in an *NPT* ensemble with periodic boundary conditions (PBC) applied in all directions. On the other hand, to assess the influence of constraints on the phase transformation, two types of cell relaxation conditions were applied: one allowing complete relaxation of the atomic positions, cell dimensions, and cell angles (denoted as unconstrained hereafter), and the other allowing only atomic positions and cell dimensions (denoted as constrained henceforth). For time integration, the Verlet algorithm was used with a time step of 2 fs. For heat treatment after the initial relaxation, the cells subjected to 1600 K were cooled to 400 K and heated back to 1600 K at rates of ±1.0 K/ps under zero external pressure. Figure 6 shows the atomic volume vs. temperature plots with (a) unconstrained and (b) constrained conditions. The phase transformation takes place from the austenite (B2) to the martensite (B19) with a decrease in temperature and vice versa. This results in an abrupt change in the atomic volume, which is consistent with the present DFT calculation. These points can be defined as austenite/martensite start/finish temperatures. The simulation results show the reversible phase transformation observed experimentally [71].

**Figure 7:**
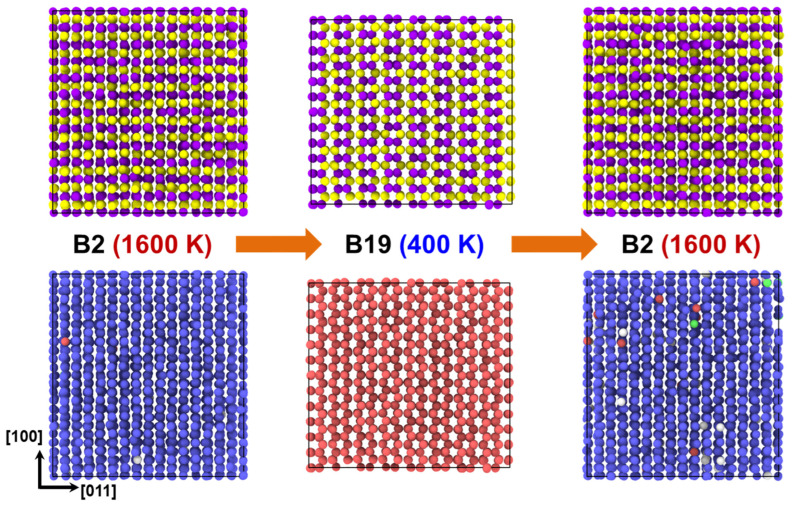
Atomic configurations of equiatomic PtTi obtained by the MD simulations performed using the present interatomic potential. The configurations at 1600 K (before cooling), 400 K (after cooling), and 1600 K (after reheating) are illustrated in sequence. In the upper row, the purple and yellow atoms denote Pt and Ti, respectively. The PTM method [95] was used to identify the B2 austenite and B19 martensite structures, whose atoms are colored blue and red, respectively, in the lower row of the figure.

For an in-depth analysis of the atomic configuration, the polyhedral template matching (PTM) method [95] was employed using the OVITO program [96]. The method helps distinguish the structures with a specific color scheme. In this case, the austenite and martensite were colored blue and red, respectively. In addition, either amorphous or free surfaces were colored gray. Figure 7 provides visual evidence of phase transformation. A clear change in the structure can be observed during the thermal process, as the figure illustrates the B2 structure at 1600 K and the B19 structure at 400 K. The characteristic feature of this phase transformation, which is the rearrangement of Pt (purple atoms) and Ti (yellow atoms) atomic positions with respect to the (110) B2 plane, can be identified easily (upper row of Figure 7). Certain atoms in the lower row of Figure 7 are distinctly colored from the surrounding atoms because of the possible thermal noise. In the current figure, the orientation relationship exhibited is ${\left(011\right)}_{B2}∥{\left(001\right)}_{B19}$ and ${\left(100\right)}_{B2}∥{\left(100\right)}_{B19}$. On the other hand, this relationship may not manifest or be observed under every condition. That is, the martensite of other multiple variants can also be formed, resulting in other orientation relationships depending on the size of the cell and boundary conditions.

**Figure 8:**
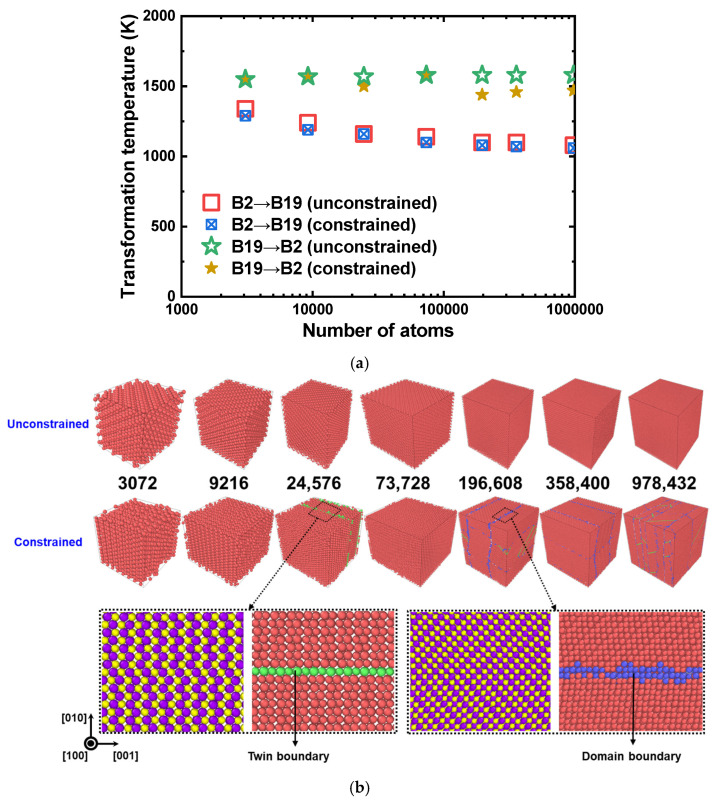
(**a**) Plot of phase transformation temperature vs. the number of atoms in a cell for the equiatomic PtTi obtained using the present interatomic potential. Results of both the unconstrained and constrained conditions are displayed. (**b**) The corresponding atomic configurations at 400 K colored according to the PTM method [95]. The number of atoms in each cell is also provided. Separate snapshots showing the twin and domain boundaries are provided below the figure. The red, blue, and atoms correspond to the B19 martensite, domain boundary, and twin boundary in the PTM snapshots, respectively. The purple and yellow atoms correspond to the Pt and Ti atoms, respectively, in the magnified snapshots.

The transformation temperature according to the atomic configurations of varying cell sizes under unconstrained and constrained conditions was examined. Figure 8a,b shows the transformation temperature plot according to the number of atoms in their configured cells and the corresponding atomic configurations, respectively. The calculated ranges of the martensite start ($M_{s}$) and austenite finish ($A_{f}$) temperatures were 1060–1340 K and 1440–1580 K, respectively, which are well within the experimentally observed values ($M_{s}\;$= 1343 K, and $A_{f\;}$= 1363 K [71]) for equiatomic PtTi SMA. In the case of the unconstrained condition, along with the increase in the number of atoms, $A_{f}$ remained almost constant, but the $M_{s}$ decreased and converged to 1080 K. The $M_{s}$ trend for the constrained case also closely matched that of the unconstrained condition, but $A_{f}$ showed no particular trend.

This phenomenon can be explained with the help of the atomic configurations illustrated in Figure 8b. Under both unconstrained and constrained cases, the starting configurations are single crystalline austenite structures at 1600 K. On the other hand, as they undergo cooling and phase transform to martensite, specific structures (cells with 24,576, 196,608, 358,400, and 978,432 atoms), in the case of constrained conditions, no longer remain single crystals. They either possess a twin boundary or domain boundary with multiple variants, or both. These defective features are formed because of the cell angle restriction, where the formation of multiple variants relieves the internal stress and energy. These features serve as the nucleation sites for austenite during heating, thereby facilitating martensite to austenite transformation at lower temperatures. The influence of those features is also reflected in the atomic volume vs. temperature plot in Figure 6. The atomic volumes, in the case of an unconstrained condition, perfectly match one another. On the other hand, the atomic volumes during cooling show a clear difference for the constrained condition structures. This is because those features occupy more volume and increase the overall atomic volume.

### 4.2. Temperature- and Stress-Induced Phase Transformation Simulation of a Nanocrystalline Equiatomic PtTi SMA

Another relatively simple yet important application of an interatomic potential is investigating the behavior and properties of the nanocrystalline structure. A cubic nanocrystalline equiatomic PtTi cell was constructed using the Voronoi construction method [97] (which generates grains with arbitrary orientations) with five grains, 29.6 nm in size, resulting in a mean grain size of 21 nm. The cell was subjected to energy minimization using the conjugate gradient method to stabilize the grain boundary regions. Before the main simulation, cell dimensions, angles, and atomic positions were relaxed at specific temperatures in an *NPT* ensemble under zero pressure and PBC.

**Figure 9:**
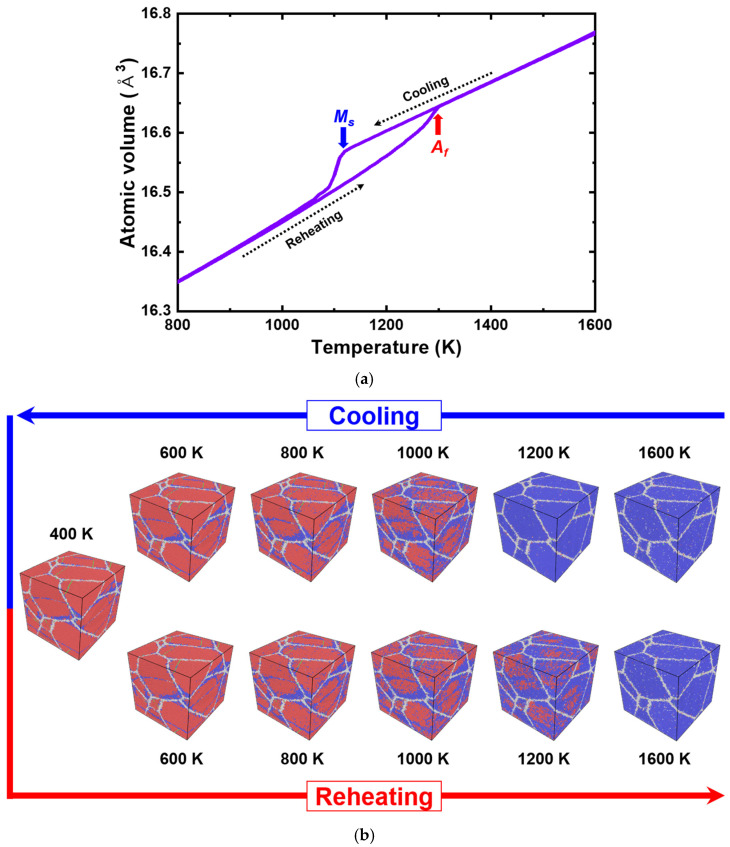
(**a**) Atomic volume vs. temperature plot of nanocrystalline equiatomic PtTi with five grains predicted using the present interatomic potential. The determined martensite start ($M_{s}$) and austenite finish ($A_{f}$) temperatures are indicated with blue and red arrows, respectively. (**b**) The corresponding atomic configurations are colored according to the PTM method [95] at regular intervals. The blue atoms correspond to the B2 austenite structure, red atoms to the B19 martensite structure, green atoms to the twin boundaries, and gray atoms to the domain or grain boundaries.

The temperature-induced phase transformation was first investigated. A similar approach to the procedure adopted in the previous section was performed. A thermal cycle from 1600 K to 400 K and back to 1600 K was applied with cooling and heating rates of 1 K/ps. Figure 9a shows the atomic volume vs. temperature plot of the nanocrystalline PtTi. The result is similar to those of the single crystals except that the austenite finish ($A_{f}$) temperature is lower for the nanocrystalline structure (1300 K). Moreover, the nanocrystalline structure transforms almost entirely to martensite with multiple variants, twins, and domain boundaries, and returns to austenite during the heat treatment cycle (Figure 9b). The atomic volume during the cooling and heating of austenite perfectly matched beyond the $A_{f}$, indicating that there is no residual martensite above that temperature. Compared to the transformation from the austenite to martensite, the reverse transformation from the martensite to austenite appears to occur more gradually over a broader range of temperatures. This indicates a relatively continuous phase transformation from austenite to martensite. A careful examination of atomic configurations revealed martensite nucleation from the interior of the grains rather than from the grain boundaries. The reverse transformation follows the same path, i.e., the austenite starts to grow from the grain and domain boundaries towards the interior regions. The calculated thermal hysteresis of this nanocrystalline PtTi structure was 183 K, with $M_{s}$ and $A_{f}$ being 1117 K and 1300 K, respectively. The value shows a notable difference from that of the unconstrained largest single crystal cell (500 K, 978,432 atoms) simulated in this work. This can be attributed to the nucleation of austenite during heating by the twin, domain boundaries and residual austenite, which ultimately lower the austenite start and finish temperatures.

The stress-induced phase transformation simulation was performed as a second application of the nanocrystalline PtTi structure. The same nanocrystalline PtTi cell used above was equilibrated at 1300 K, which is the $A_{f}$ temperature determined from the temperature-induced phase transformation simulation to maintain the austenite phase under a zero-stress initial state. Independent stress-controlled uniaxial tensile and compressive cyclic loading/unloading simulations were performed at this constant temperature. A stress rate of 12.5 MPa/ps was used with a maximum stress limit of 1200 MPa. During the simulation, the pressure perpendicular to the loading directions was set to zero, and the cell angles between those directions were allowed to relax.

**Figure 10:**
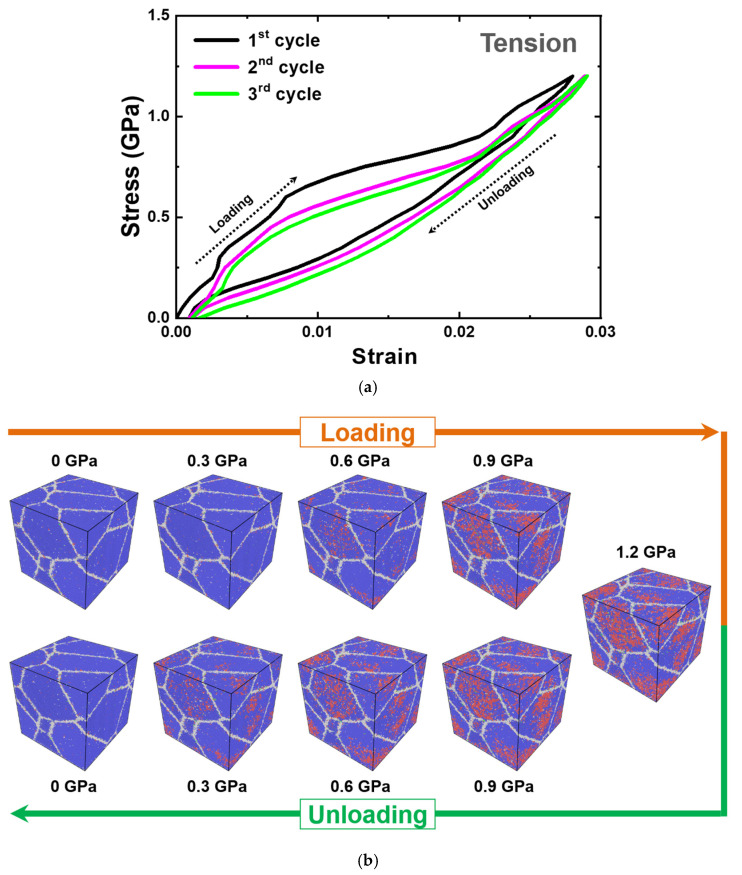
(**a**) Tensile stress–strain curve of the nanocrystalline equiatomic PtTi with 5 grains simulated at 1300 K. (**b**) The corresponding atomic configurations of the third cycle colored according to the PTM method [95] at regular intervals. The blue atoms correspond to the B2 austenite structure, red atoms to the B19 martensite structure, green atoms to the twin boundaries, and gray atoms to the domain or grain boundaries.

Figure 10a shows the tensile stress–strain curve of nanocrystalline PtTi performed for three cycles. As the nanocrystalline PtTi cell is loaded, it deforms elastically first as any other material. Subsequently, the slope changes as the cell is strained beyond 0.0075. This is because of the stress-induced phase transformation from austenite to martensite. As the martensite fraction reaches the maximum under the stress-induced phase transformation for this given temperature, another change in slope occurs towards a higher value. The cell is unloaded after reaching the peak stress condition. The unloading curve does not follow the loading curve, producing a hysteresis loop. A noteworthy attribute of the unloading curve is that the curve does not exhibit a clear phase transformation interval, and the change in slope is smoother. This is indicative of a relatively continuous austenite-to-martensite phase transformation observed in the temperature-induced phase transformation thermal cycle atomic volume vs. temperature plot (Figure 9a). Another notable feature is the residual strain accumulated after the first cycle. This can be attributed to the plastic deformation of the grain boundary regions instead of a dislocation slip or to the presence of the residual martensite, as reported elsewhere on a nanocrystalline NiTi SMA MD simulation [48]. As shown in Figure 10b, the atomic configuration, after fully unloading, does not contain residual martensite. On the other hand, this residual strain can be alleviated through a process known as pre-training, which subjects the SMA to cyclic loading. This has implications for the stabilization of the hysteresis response, which can also be verified from Figure 10a.

One more similar aspect of the stress-induced phase transformation and temperature-induced phase transformation is the nucleation and growth of martensite. Figure 10b shows the atomic configurations at even intervals from the third loading cycle. The martensite nucleates from the interior of the grains and grows towards the grain boundaries. On the other hand, the low fraction of stress-induced martensite, however, is evidently visible. This can be elucidated from the anisotropy associated with the stress-induced transformation and the relative stability of austenite and martensite. The anisotropy becomes involved because of the fixed loading direction and the multi-crystalline nature of the cell. If a grain is not oriented preferentially in a pre-defined loading direction, even a high loading might not be able to entirely transform it into martensite. The reason for the absence of twins and multiple variants under stress could again be the non-preferential orientation of grains. In addition, the martensitic transformation is fundamentally based on the relative stability of austenite and martensite under a given condition and is highly sensitive to temperature. If stress is applied, it can momentarily enhance the stability of martensite over the austenite. On the other hand, only a partial region of the grain can transform if the temperature is too high, such that the stress applied cannot significantly influence the martensite stability.

**Figure 11:**
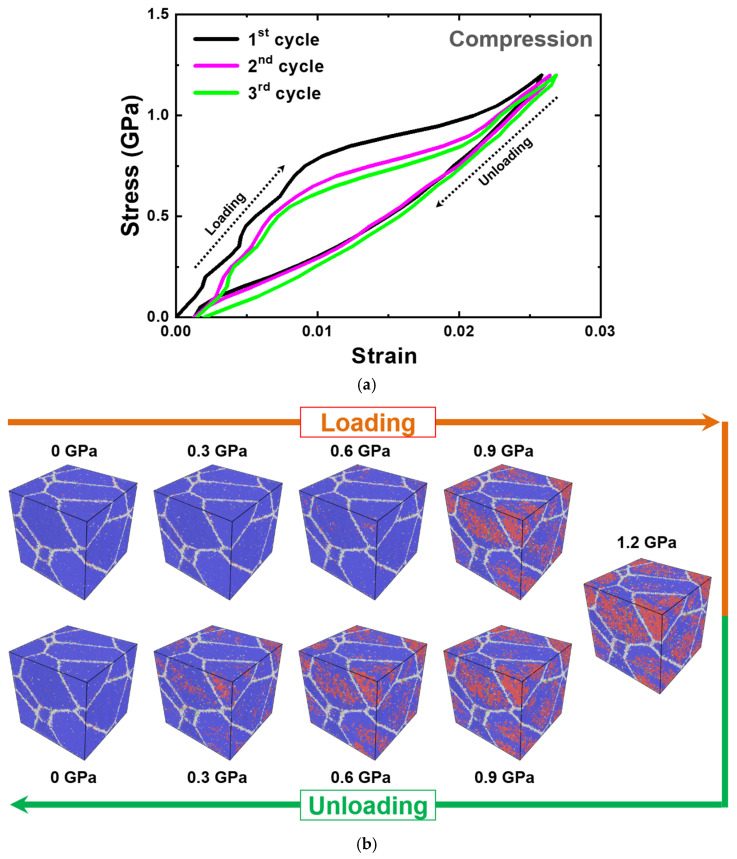
(**a**) Compressive stress–strain curve of the nanocrystalline equiatomic PtTi with five grains simulated at 1300 K. (**b**) The corresponding atomic configurations of the third cycle colored according to the PTM method [95] at regular intervals. The blue atoms correspond to the B2 austenite structure, red atoms to the B19 martensite structure, green atoms to the twin boundaries, and gray atoms to the domain or grain boundaries.

The compressive stress–strain curve of the same nanocrystalline PtTi structure also showed similar results, such as the residual strain, influence of pre-straining, and continuous phase transformation from martensite to austenite, as illustrated in Figure 11. However, higher phase transformation stress is reached compared to the tensile simulation case. This can be attributed to the influence from the crystallography of the martensitic transformation and the transformation anisotropy [98,99]. The differences extend to the fraction of martensite formed, the nucleation sites, and possibly the variants caused by the aforementioned anisotropy and influence of the grain orientation.

The above simulations employed the developed interatomic potential. They demonstrated the martensitic transformation under thermal and mechanical loading conditions to effectively show the shape memory effect and the superelasticity of the nanocrystalline equiatomic PdTi SMA. However, it should be kept in mind that the mechanical responses and the resultant properties also depend on various conditions, such as the orientation, shape, and size of the grains. In addition, the role of dislocation is not to be neglected because its activity at high temperatures could be a serious issue, affecting the characteristics and responses of SMAs, leading to incomplete shape recovery as reported elsewhere [100]. A study on the effects of the dislocations was not carried out because it was beyond the scope of this work. A study on the dislocation influence of equiatomic PtTi SMA and other compositions using the present interatomic potential could be a topic of future work. Finally, an extension of the present potential may be carried out to construct a ternary interatomic potential for studies on more diverse compositions and structures.

## 5. Conclusions

A 2NN MEAM model-based Pt–Ti binary interatomic potential was developed to fit the parameters of the equiatomic PtTi configurations for simulating martensitic transformations and provide accurate predictions of its related phenomena and properties to analyze the PtTi SMA characteristics and attributes. The present interatomic potential could predict the properties of various intermetallic compounds and alloys with different compositions and structures with good accuracy and transferability. The unconstrained and constrained conditions of the temperature-induced martensitic transformation of single crystalline MD simulations using the present interatomic potential showed that the twin and domain boundary could form to accommodate the constraint on the grain (a constraint on the cell angle in this work), which results in a premature martensite to austenite phase transformation due to heterogeneous nucleation. Another analysis of the temperature- and stress-induced martensitic transformation of nanocrystalline MD simulations reproduced the shape memory effect and superelasticity phenomena of polycrystalline SMA. The role of anisotropy and grain orientation on the fraction of martensite, formation of twins, and domain boundaries was presented from the difference in atomic configurations of the temperature- and stress-induced martensitic transformations. The Pt–Ti binary interatomic potential developed in this work can be utilized to study the microstructural changes under various temperature conditions associated with the shape memory aspect of equiatomic PtTi and other alloy compositions and structures.

## Figures and Tables

**Table 1:** Finalized 2NN MEAM interatomic potential parameters for the pure Pt and Ti. The units of cohesive energy ($E_{c}$), equilibrium nearest-neighbor distance ($r_{e}$), and bulk modulus (B) are eV/atom, Å, and 10^12^ dyne/cm^2^, respectively. Other parameters are unitless. The reference structures used are fcc and bcc for pure Pt and Ti, respectively.

	$E_{c}$	$r_{e}$	B	A	*β* ^(0)^	$\beta^{\left(1\right)}$	$\beta^{\left(2\right)}$	$\beta^{\left(3\right)}$	$t^{\left(1\right)}$	$t^{\left(2\right)}$	$t^{\left(3\right)}$	$C_{\min}$	$C_{\max}$	d
Pt	5.77	2.770	2.8838	0.93	5.20	4.60	0.20	0.60	1.50	2.70	6.70	0.45	2.91	0.05
Ti ^a^	4.75	2.850	1.0735	0.24	2.20	3.00	4.00	3.00	−18.0	−32.0	−44.0	0.25	1.58	0.00

^a^ Ref. [72].

**Table 2:** Finalized 2NN MEAM interatomic potential parameter for the binary Pt–Ti system. The parameters with units are the enthalpy of formation of the reference structure (B2 PtTi) ($\Delta E_{f}$, eV/atom), the equilibrium nearest-neighbor distance ($r_{e}$, Å), and the bulk modulus (B, 10^12^ dyne/cm^2^). Detailed descriptions of the interatomic potential parameters for binary alloys can be found elsewhere [70,84].

Parameter	Optimized Value
$\Delta E_{f}(0.5E_{c}^{fcc\;Pt}+0.5E_{c}^{bcc\;Ti}-E_{c}^{B2\;PtTi})$	−0.8484
$r_{e}$	2.755
B	1.84253
d	0.5$d^{Pt}\;$+ 0.5$d^{Ti}$
$\rho_{0}^{Pt}$:$\rho_{0}^{Ti}$	1:1
$C_{\min}^{Pt-Ti-Pt}$	0.09
$C_{\min}^{Ti-Pt-Ti}$	0.64
$C_{\min}^{Pt-Pt-Ti}$	0.36
$C_{\min}^{Ti-Ti-Pt}$	1.60
$C_{\max}^{Pt-Ti-Pt}$	2.80
$C_{\max}^{Ti-Pt-Ti}$	1.44
$C_{\max}^{Pt-Pt-Ti}$	2.00
$C_{\max}^{Ti-Ti-Pt}$	2.00

**Table 3:** Physical properties and various energies of pure Pt obtained from the experiment, present DFT calculations (LDA, and GGA), and previous [69] and present 2NN MEAM interatomic potentials. Cohesive energy $(E_{c}$), lattice parameter (a), bulk modulus (B), elastic constants ($C_{11}$, $C_{12}$, and $C_{44}$), difference in structural energy (ΔE), energy of vacancy formation ($E_{f}^{\mathrm{vac}}$), energy of vacancy migration ($E_{m}^{\mathrm{vac}}$), activation energy of vacancy diffusion ($Q^{\mathrm{vac}}$), and surface energy ($E_{surf}$) are compared. The respective units are provided in the table.

Property	Exp.	DFT (LDA) ^d^	DFT (GGA) ^d^	2NN MEAM [Lee]	2NN MEAM [This Work]
$E_{c}$ (eV/atom)	5.77 ^a^	7.044	5.526	5.770	5.770
a (Å)	3.924 ^b^	3.897	3.977	3.917	3.917
B (10^12^ dyne/cm^2^)	2.884 ^c^	3.056	2.471	2.8838	2.8816
$C_{11}$ (10^12^ dyne/cm^2^)	3.580 ^c^			3.581	3.638
$C_{12}$ (10^12^ dyne/cm^2^)	2.536 ^c^			2.535	2.507
$C_{44}$ (10^12^ dyne/cm^2^)	0.774 ^c^			0.775	0.946
$\Delta E_{\mathrm{fcc}\to \;\mathrm{bcc}}$ (eV/atom)		0.113	0.092	0.28	0.121
$\Delta E_{\mathrm{fcc}\to \mathrm{hcp}}$ (eV/atom)		0.063	0.055	0.02	0.036
$E_{f}^{\mathrm{vac}}$ (eV)	1.35, 1.5 ^c^	0.756	0.561	1.50	1.816
$E_{m}^{\mathrm{vac}}$ (eV)		1.287	1.106	0.20	1.113
$Q^{\mathrm{vac}}$ (eV)	2.64 ^c^	2.044	1.666	2.70	2.928
$E_{\mathrm{surf}}^{\left(100\right)}$ (erg/cm^2^)		2394	1823	2288	1560
$E_{\mathrm{surf}}^{\left(110\right)}$ (erg/cm^2^)		2477	1868	2328	1714
$E_{\mathrm{surf}}^{\left(111\right)}$ (erg/cm^2^)		1978	1475	1710	1314

^a^ Ref. [83]. ^b^ Ref. [87]. ^c^ Ref. [69] and the references therein. ^d^ Present DFT calculations.

**Table 4:** Thermal properties associated with the melting of pure Pt obtained from the experiment, previous [69] and present interatomic 2NN MEAM interatomic potential. The target properties are the melting temperature ($T_{m}$), enthalpy of melting ($\Delta H_{m}$), and volume change upon melting ($\Delta V_{m}/V_{\mathrm{solid}}$). The respective units are provided in the table.

Property	Exp. ^a^	2NN MEAM [Lee]	2NN MEAM [This Work]
$T_{m}$ (K)	2042	2374	2160
$\Delta H_{m}$ (kJ/mol)	22.2	33.2	22.9
$\Delta V_{m}/V_{\mathrm{solid}}$ (%)		9	6.2

^a^ Ref. [69] and the references therein.

**Table 5:** Comparison of the physical properties of intermetallic compounds, viz., lattice parameters (a, b, and c), atomic volume (Ω), bulk modulus (B), and enthalpy of formation ($\Delta E_{f}$) obtained from the experiment, present DFT calculation (LDA and GGA), and present interatomic potential. The respective units are provided in the table.

Composition	Structure (Space Group)	Property	Exp.	DFT (LDA) ^c^	DFT (GGA) ^c^	2NN MEAM
Pt_1_Ti_1_	B2 ($Pm\bar{3}m$)	a (Å)	3.172 ^a^	3.118	3.181	3.181
		Ω (Å^3^)		15.14	16.08	16.10
		B (10^12^ dyne/cm^2^)		2.198	1.846	1.857
		$\Delta E_{f}$ (eV/atom)		−0.786	−0.794	−0.791
	B19 (Pcmm)	a (Å)	4.592 ^a^	4.541	4.632	4.552
		b (Å)	2.761 ^a^	2.719	2.777	2.911
		(Å)	4.838 ^a^	4.792	4.882	4.793
		Ω (Å^3^)		14.79	15.70	15.88
		B (10^12^ dyne/cm^2^)		2.341	1.983	1.892
		$\Delta E_{f}$ (eV/atom)		−0.960	−0.931	−0.834
Pt_8_Ti_1_	$D1_{a}$ (I4/m)	a (Å)	8.312 ^a^	8.256	8.426	8.413
		c (Å)	3.897 ^a^	3.866	3.942	3.860
		B (10^12^ dyne/cm^2^)		2.977	2.433	2.644
		$\Delta E_{f}$ (eV/atom)		−0.472	−0.453	−0.259
Pt_3_Ti_1_	$D0_{24}$ $(P6_{3}/mmc$)	a (Å)		5.508	5.615	5.481
		c (Å)		4.432	4.522	4.527
		B (10^12^ dyne/cm^2^)		2.835	2.334	2.362
		$\Delta E_{f}$ (eV/atom)		−0.883	−0.851	−0.617
Pt_3_Ti_1_	$L1_{2}$ $(Pm\bar{3}m$)	a (Å)	3.916 ^a^	3.877	3.952	3.943
		B (10^12^ dyne/cm^2^)		2.840	2.361	2.418
		$\Delta E_{f}$ (eV/atom)		−0.891	−0.860	−0.618
Pt_3_Ti_1_	$(P\bar{3}m1$)	a (Å)		5.484	5.591	5.582
		c (Å)		15.659	15.962	15.922
		B (10^12^ dyne/cm^2^)		2.834	2.361	2.399
		$\Delta E_{f}$ (eV/atom)		−0.894	−0.863	−0.617
Pt_5_Ti_3_	(Ibam)	a (Å)	5.441 ^b^	5.398	5.492	5.550
		b (Å)	8.169 ^b^	8.053	8.261	8.095
		c (Å)	10.953 ^b^	10.874	11.066	11.107
		B (10^12^ dyne/cm^2^)		2.541	2.128	2.014
		$\Delta E_{f}$ (eV/atom)		−0.973	−0.952	−0.658
Pt_1_Ti_3_	A15 $(Pm\bar{3}n$)	a (Å)	5.0335 ^a^	4.936	5.044	5.089
		B (10^12^ dyne/cm^2^)		1.861	1.584	1.505
		$\Delta E_{f}$ (eV/atom)		−0.700	−0.652	−0.545

^a^ Ref. [85] and the references therein. ^b^ Ref. [91]. ^c^ Present DFT calculation.

**Table 6:** Comparison of the predicted energies of binary solid solutions, solute in Pt-rich fcc and Ti-rich hcp structures, obtained from the present DFT calculation (LDA and GGA) and the present interatomic potential. Fcc Pt and hcp Ti were taken as the reference structures for the calculation of dilute heat of solution ($E_{d}^{sol}$). The first and second nearest-neighbor vacancy–solute binding energy ($E_{b}^{vac-sol}$) and solute–solute binding energy ($E_{b}^{sol-sol}$) in Pt-rich fcc are denoted by “1NN” and “2NN”, respectively. The same binding energies, along with the solute migration energy ($E_{m}^{sol}$) in Ti-rich hcp, are denoted using “in” and “out” for the in-basal and out-of-basal configurations, respectively. The negative sign of binding energy indicates a repulsive interaction.

Structure	Property (eV)	DFT (LDA) ^a^	DFT (GGA) ^a^	2NN MEAM
Pt-rich fcc	$E_{d}^{Ti}$	−4.0050	−3.8830	−2.1650
	$E_{b}^{vac-Ti}$ *(1NN)*	−0.1942	−0.1807	−0.1203
	$E_{b}^{vac-Ti}$ *(2NN)*	−0.0100	−0.0084	−0.0027
	$E_{b}^{Ti-Ti}$ *(1NN)*	−0.4696	−0.4329	−0.4352
	$E_{b}^{Ti-Ti}$ *(2NN)*	−0.0019	−0.0031	0.1944
	$E_{m}^{Ti}$	1.5285	1.3640	2.2510
Ti-rich hcp	$E_{d}^{Pt}$	−1.9244	−1.7236	−2.1066
	$E_{b}^{vac-Pt}$ *(in)*	−0.1120	0.0273	−0.1499
	$E_{b}^{vac-Pt}$ *(out)*	0.0057	0.1101	−0.1795
	$E_{b}^{Pt-Pt}$ *(in)*	−0.1957	−0.0692	−0.3280
	$E_{b}^{Pt-Pt}$ *(out)*	−0.0952	0.0147	−0.4192
	$E_{m}^{Pt}$ *(in)*	0.7367	0.7840	2.5094
	$E_{m}^{Pt}$ *(out)*	1.0188	0.9449	2.6509

^a^ Present DFT calculation.

## Data Availability

The data that support the finding of this study is available from the corresponding author (email: wonsko@inha.ac.kr) upon reasonable request.
